# A novel fluorescent pH probe for expression in plants

**DOI:** 10.1186/1746-4811-2-7

**Published:** 2006-04-06

**Authors:** Alexander Schulte, Inken Lorenzen, Markus Böttcher, Christoph Plieth

**Affiliations:** 1 Zentrum für Biochemie und Molekularbiologie, Universität Kiel, Am Botanischen Garten 9, 24118 Kiel, Germany; 2 Biochemisches Institut, Universität Kiel, Rudolf-Höber-Str. 1, 24098 Kiel, Germany; 3 Botanisches Institut, Universität Kiel, Am Botanischen Garten 9, 24118 Kiel, Germany; 4 Institut für Physiologie, Universität zu Lübeck, Ratzeburger Allee 160, 23538 Lübeck, Germany

## Abstract

**Background:**

The pH is an important parameter controlling many metabolic and signalling pathways in living cells. Recombinant fluorescent pH indicators (pHluorins) have come into vogue for monitoring cellular pH. They are derived from the most popular *Aequorea victoria *GFP (*Av-*GFP). Here, we present a novel fluorescent pH reporter protein from the orange seapen *Ptilosarcus gurneyi *(*Pt-*GFP) and compare its properties with pHluorins for expression and use in plants.

**Results:**

pHluorins have a higher pH-sensitivity. However, *Pt-*GFP has a broader pH-responsiveness, an excellent dynamic ratio range and a better acid stability. We demonstrate how *Pt*-GFP expressing *Arabidopsis thaliana *report cytosolic pH-clamp and changes of cytosolic pH in the response to anoxia and salt-stress.

**Conclusion:**

*Pt-*GFP appears to be the better choice when used for *in vivo-*recording of cellular pH in plants.

## Introduction

Fluorescent proteins have revolutionized the understanding of cellular event cascades, signal transduction, and structure dynamics [1,2]. The green fluorescent protein from *Aequorea victoria *(*Av*-GFP) is the most popular species used by scientists to date. *Av*-GFP and its corresponding cDNA has been altered many times to give fluorescent proteins of higher quantum efficiency, different spectral characteristics, less temperature sensitivity, improved solubility, and higher expression levels in other organisms [e.g. [3,4]]. Enhanced variants of *Av-*GFP are frequently used to decorate cellular structures and proteins in order to observe shape, location and dynamics *in vivo *[e.g. [5-12]] or to visualize gene expression and/or activity of promoters or enhancers [13].

One of the advantages of GFPs is their ability to be engineered to indicators for cellular signal transduction studies [e.g. [14-16]]. Engineered GFPs have been used in plants to report cellular concentrations of Ca^2+^, H^+^, Cl^-^, and NO_3_^- ^[e.g. [17-23]]. No loading of the indicator is necessary with GFP-based probes and they can be precisely targeted to almost any organelle, compartment or tissue in question [e.g. [8,9,11,12,24]]. This potentially makes GFP-derived probes superior to small molecular weight fluorescent dyes used as ion indicators provided they can replicate the sensitivity and responsiveness of these probes.

In particular two pH-sensitive variants of *Av*-GFP (so-called pHluorins) have been engineered [25]. These two reporters are called 'ratiometric' and 'ecliptic' pHluorin. They both allow ratiometric *in vivo*-pH recording. Ratiometric pHluorin is a double excitation indicator whereas ecliptic pHluorin has to be used in the double emission mode [21]. The amendments necessary for sufficient expression of *Av*-GFPs in plants (i.e. removal of the cryptic intron, changes to *A. thaliana *codon usage and improvement of solubility) have been combined with the properties of pHluorins. The resulting pH indicators have been successfully expressed and used in *Arabidopsis *[21,26,27].

However, more and more fluorescent proteins (FPs) from other marine organisms are being discovered with other interesting properties [28-35]. Some of these newly discovered FPs unveil advantages when compared with *Av*-GFP variants. We have expressed the GFP from the orange seapen (*Ptilosarcus gurneyi*) in bacteria and plants. Here, we compare ratiometric properties of *Pt*-GFP with those of pHluorins and also with conventional fluorescent dyes often used for ratiometric pH measurements *in vivo*.

The spectrum of a fluorescent ratiometric indicator is in a first approach mainly the sum or overlap of two spectra [36]: First, the spectrum of the free indicator (in case of pH indicators the de-protonated form) and second the spectrum of the bound (protonated) indicator molecules. There are further two different effects on the fluorescence of pH indicators which need to be distinguished when the pH is lowered: First a fluorescence quenching at all wavelengths. Second, the spectral disproportionation (i.e. the attenuation of the spectrum from de-protonated indicator for the benefit of the fluorescence spectrum formed by the protonated molecules). Ratiometric fluorescence measurements have been established to cancel down all side-effects based on variations in indicator concentration, illumination intensity, detector sensitivity etc. [37,38]. So, only the second effect, namely spectral disproportionation is essential and relevant for ratiometry and, in theory, the ratio solely correlates with the analyte concentration (here [H^+^]). A quenching at all wavelengths can be considered an apparent decrease in indicator concentration and is thus irrelevant for ratiometry.

Here all spectra are presented normalized by their area for three reasons: First, this is a way to uncover all spectral effects relevant for ratiometric measurements. Second, it is the optimal way to present the potential capabilities of the indicator (i.e. best pair of wavelengths, dynamic ratiometric change etc.) when intended for ratiometry and/or ratio imaging. Third, it does not require an *a-priori-*knowledge of the isosbestic point.

By calculating the so-called minimax spectrum (i.e. the spectrum that is obtained when the minimum fluorescence is substracted from the maximum fluorescence at each wavelength within a scanned set of spectra) it is possible to derive two important indicator characteristics: First, the real isosbestic point is the wavelength where the fluorescence is independent from spectral disproportionation. Here, the minimax spectrum, in theory, is zero with discontinuous slope, but, in practice, approaches a minimum close to zero. Second, the sensitivity of the indicator which is defined here as the integral of the minimax spectrum. The sensitivity is a number in the range between zero and two. It is two when there is no spectral overlap of the protonated and the de-protonated form of the indicator (ideal ratiometric indicator). It approaches zero when the indicator is less suitable for ratiometry. Consequently, the normalized spectra give the real isosbestic point while in the raw data spectra (not given here) this point is shifted by the superposed quench effect and is then distinguished here as 'apparent' isosbestic point.

## Results & Discussion

### Spectral properties of recombinant pH indicators

**Figure 1:**
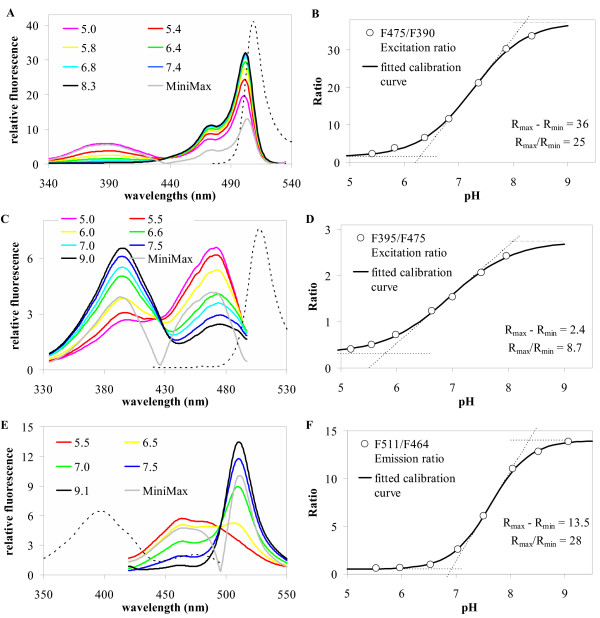
Fluorescence properties of genetically encoded pH-indicators. Spectra taken at different pHs are given for *Pt*-GFP (**A**), ratiometric pHluorin (**C**) and ecliptic pHluorin (**E**). Corresponding ratio curves are on the right hand side (**B, D, F**). **A: **Excitation spectra of *Pt*-GFP at λ_em _= 540 nm. The dotted line represents the emission spectrum (λ_ex _= 470 nm) corresponding to pH = 7.4. **B: **Dependency of fluorescence excitation ratios R(475ex/390ex; 540em) of *Pt*GFP on pH. **C: **Excitation spectra of ratiometric pHluorin at λ_em _= 508 nm. The dotted line represents the emission spectrum (λ_ex _= 390 nm) corresponding to pH 7.5. **D: **Dependency of fluorescence excitation ratios R(395ex/475ex; 508em) of ratiometric pHluorin on pH. **E: **Emission spectra of ecliptic pHluorin taken at different pH (λ_ex _= 400 nm). The dotted line represents the excitation spectrum (λ_em _= 508 nm) corresponding to pH 7.5. **F: **Dependency of fluorescence excitation ratios R(400ex; 511em/464em) of ecliptic pHluorin on pH. All spectra are normalized by their area. The grey curve in each set of spectra (**A, C, E**) represents the corresponding minimax spectrum (maximum fluorescence difference for each wavelength). Its minimum designates the isosbestic point. Ratio data (**B, D, F**) were fitted with a sigmoidal Boltzmann fit.

Ratiometric fluorescent indicators are typically characterized by a set of fluorescence spectra taken under different analyte concentrations (here: different pH values) and otherwise identical conditions. The normalized spectra (coloured curves in Figure 1) allow to calculate the minimax spectrum (grey lines in Figure 1) and to extract a number of characteristic parameters which give clues about signal quality, signal-to-noise ratio, sensitivity, and the best application range of the indicator and about the optimal optical setting when used *in vivo*. The parameters most relevant for practical work are:

1 The isosbestic points λ_iso _(precisely, the isoexcitation points in case of a double excitation probe or the isoemission points in case of a double emission probe) are the wavelengths where the fluorescence is independent of the indicated analyte (ion) concentration. Here, the real isosbestic point is distinguished from the apparent isosbestic point.

2 Spectral peaks or shoulders left (λ_1_) and right (λ_2_) of the isosbestic point λ_iso _which vary in opposite direction when the analyte concentration is changed.

3 The maximum fluorescence wavelength (λ_max_) is the peak in the emission spectrum in case of a double excitation indicator (such as ratiometric pHluorin and *Pt-*GFP) and the maximum in the excitation spectrum in case of a double emission indicator (such as ecliptic pHluorin).

**Figure 2:**
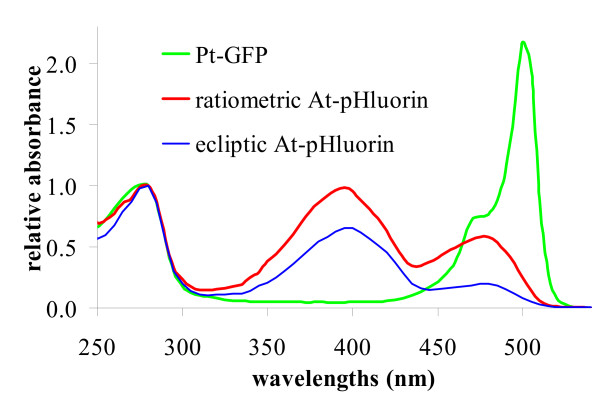
Absorption spectra of pH sensitive GFPs. The spectra were taken in phosphate buffer (pH = 7.4) and normalized by A280. The absorption spectra coincide with the excitation spectra shown in Figures 1.

4 The Stokes-shift of a fluorescent probe is defined by the difference between the wavelength of its absorption peak (Figure 2) and the wavelength of its emission peak (Figure 1).

5 The maximum and minimum ratios, R_max _and R_min _(i.e. the ratios taken in the absence of the analyte or when the indicator is saturated with analyte).

6 The apparent pK (i.e. midpoint of the calibration curve where the ratio reaches half maximum between R_min _and R_max_) is dependent on the ratiometric wavelengths chosen (see supplemental data in additional file 1). Ideally, the apparent pK is about the dissociation constant which is defined by the analyte concentration where the indicator is half-saturated.

**Figure 3:**
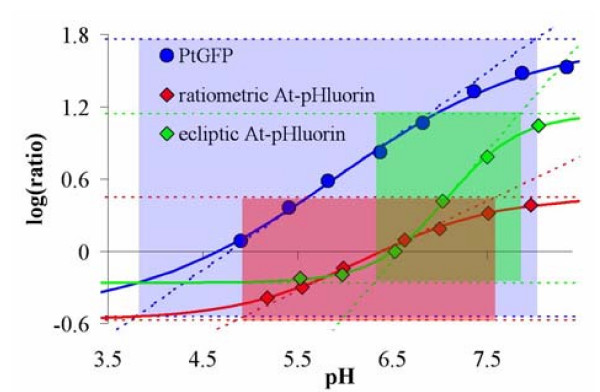
Comparison of all three recombinant pH-probes on a log-log scale. The areas of best responsiveness are highlighted by coloured rectangles: ecliptic pHluorin in red (emission ratio R_em _= F511/F465 at λ_ex _= 400 nm); ratiometric pHluorin in green (excitation ratio R_ex _= F390/F475 at λ_em _= 508 nm), and *Pt*-GFP in blue (excitation ratio R_ex _= F475/F390 at λ_em _= 540 nm).

7 The useful concentration range (x-axis range) in which the indicator is reasonably applied and where the logarithm of the ratio depends approximately linear on the logarithm of analyte concentration. In the case of pH-indicators this range is given by the pH values where the straight line through the midpoint of the logarithmic calibration curve intersects with log(R_max_) and log(R_min_) (Figure 3).

8 The sensitivity S of the indicator is defined here (see introduction section) by the area or integral of the minimax spectrum. The sensitivity is excellent when S > 0.5 (Table 1) and it is negligible when S < 0.1 (Table 4).

**Table 1:** Spectral characteristics of recombinant pH-probes. The real isosbestic point is derived from normalized spectra whereas the apparent isosbestic point is from the raw data. The Stokes-shifts are defined here by the difference between the wavelength of the major (and the minor) absorption peak and the wavelength of the emission peak. Index 'x' of S designates sensitivity calculated from excitation spectra, and index 'm' values are calculated from emission spectra. 'em', 'ex', and 'abs' designate wavelengths in the emission spectrum, the excitation spectrum, and in the absorption spectrum, respectively.

	**GFP-species**		
**parameter**	**ratiometric pHluorin**	**ecliptic pHluorin**	***Pt*-GFP**
real isosbestic point λ_iso_	426 nm (ex)	495 nm (em)	437 nm (ex)
apparent isosbestic point	428 nm (ex)	489 nm (em)	430 nm (ex)
left peak WL (λ_1 _< λ_iso_)	395 nm (ex, abs)	464 nm (em)	390 nm (ex)
right peak WL(λ_2 _> λ_iso_)	475 nm (ex)	511 nm (em)	502 nm (ex)
Stokes-shift (λ_em _- λ_abs_)	113 nm (33 nm)	114 nm (35 nm)	6 nm
maximum WL (λ_max_)	508 nm (em)	397 nm (ex, abs)	508 nm (em)
R_max _(F(λ_1_)/F(λ_2_))	2.75	14	107
R_min _(F(λ_1_)/F(λ_2_))	0.3	0.5	4.2
R_max _- R_min_	2.44	13.5	103
R_max_/R_min_	8.7	27	26
apparent pK (λ_1_; λ_2_)	6.9	7.6	7.3
Responsiveness (Δlog(R)·ΔpH)	2.9	2.0	**9.8**
pH sensitivity (S_x_)	**0.77**	0.41	**0.69**
pH sensitivity (S_m_)	0.34	**1.05**	0.09

**Table 4:** Comparison of camera exposure times needed for ratiometric pHluorin and *Pt*-GFP when excitation ratios R(F475; F390) are taken in plants under otherwise identical settings.

	GFP-species		
parameter	ratiometric pHluorin	*Pt*-GFP
390 nm	180	300
475 nm	150	100

The parameters for the three pH-reporter proteins discussed here, namely *Pt*-GFP, ratiometric, and ecliptic pHluorin are given for comparison in Tables 1 and 2. Spectra and calibration curves are depicted in Figure 1. Parameters of some chemical fluorescent dyes often used as ratiometric *in vivo *pH indicators are given for further comparison in an additional file (Table).

**Table 2:** Comparison of ratiometric pHluorin an *Pt*-GFP when excitation ratios R(F475; F390) are taken. The optimal pH-range is calculated here from a double log-plot as shown in Figure 3

	GFP-species		
parameter	ratiometric pHluorin	*Pt*-GFP
R_max_	2.65	37.4
R_min_	0.3	1.5
R_max_/R_min_	8.8	25
R_max _- R_min_	2.35	36
apparent pK (390 nm; 475 nm)	6.9	7.3
optimal pH-range	4.8 < pH < 7.6	3.8 < pH < 8.2

When doing ratio imaging, a maximum dynamic fluorescence ratio range (i.e. a maximum fold fluorescence ratio increase R_max_/R_min_) is desired in order to gain an optimal signal to noise ratio. However, the experimental conditions – in particular the spectral characteristics of the available filter set and/or dichroic mirror – are often not optimised for the ratiometric probe in use or the Stokes-shift of the indicator is too small for reliably separating fluorescence emission from excitation light. In case of *Pt-*GFP, for instance, the Stokes-shift is just 6 nm (i.e. right excitation peak λ_2 _= 502 nm; maximum emission λ_max _= 508 nm; see Table 1). This is too close to be separated by conventional microscopic filter sets. Hence, wavelengths other than those giving the maximum dynamic ratio range are compulsorily chosen. Thereby, it should be kept in mind that responsiveness and midpoint of the calibration curve (apparent pK) depend on the two wavelengths chosen for ratio measurements. This effect is demonstrated as supplemental data in additional file 1 (Figure S1, Table S2).

For *in vivo *comparison of ratiometric pHluorin and *Pt-*GFP we used the F475 nm/F390 nm pair for excitation with an emission range between 510 nm ≤ λ_em _≤ 560 nm. In Table 2 the optical properties for this particular pair are listed for comparison.

Both, ratiometric and ecliptic pHluorins do not differ significantly in their spectra when other proteins are fused to the N- and/or the C-terminus [21]. This is also true for *Pt-*GFP (unpublished observations). This property is important since fusions with transit or signal peptides are often used to specifically target the indicator to subcellular locations.

For direct comparison of all three pH-probes the ratios where plotted on a log-log-scale (Figure 3). This allows to determine the area of best indicator responsiveness (dynamic ratio range *vs*. dynamic pH range). The diagram (Figure 3) clearly shows that *Pt*-GFP has the best responsiveness (Δlog(R)·pH = 9.8) and the broadest pH-application range. The responsiveness is lower with ratiometric pHluorin (2.9) and ecliptic pHluorin (2.0). *Pt-*GFP responsiveness also exceeds that of conventional pH-indicators (Table S1 in additional file). However, *Aequorea *GFPs have a better sensitivity than *Pt-*GFP (Table 1) but the sensitivities of all three recombinant indicators are of similar magnitude (0.65 < S < 1) when compared with conventional dyes (Table S1).

### pH-stability of GFPs

The fluorescence quench at all wavelengths by low pH has already been mentioned above. This is due to reversible protonation of *Av-*GFPs in the range 7 > pH > 5 and by irreversible conformational changes leading to protein instability in the range pH < 5 [39,40]. The latter effect is undesirable when GFPs are used as pH-probes in plants. The apoplast of plant cells is usually acidic (pH < 6.5) [21,41,42], and also some vacuoles have low pH. Thus, cytoplasmic pH changes can be drastic in plants (see e.g. *in vivo *experiments below). Therefore it is good to have a pH indicator with high acid stability and ratiometric responsiveness in the lower pH range.

**Figure 4:**
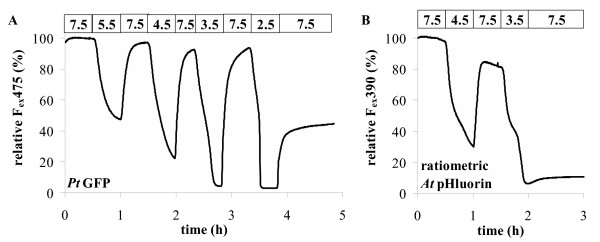
pH-stability of *Pt*-GFP (**A**) and ratiometric pHluorin (**B**). Agarose beads decorated with GFP and sandwiched between sheets of cellophane were dialysed against buffers of different pH as indicated by the top bars of the graphs and fluorescence (F_em _= 535 ± 25 nm) was recorded with a fluorescence microscope. (Buffer composition: 50 mM Hepes, 50 mM Mes, 200 mM NaCl adjusted with NaOH or HCl to the desired pH).

To quantify indicator stability at low pH we recorded GFP fluorescence and its reversibility during low pH-treatment (Figure 4). GFPs from *Aequorea victoria *(*Av*-GFPs) do not recover from a treatment with pH lower than 4 (Figure 4B) whereas *Ptilosarcus *GFP is more stable at low pH and does recover to approx. 40% even after 30 min at pH = 2.5 (Figure 4A). This acid stability of *Pt*-GFP is a special advantageous feature that will allow to overcome difficulties that have been experienced when *Av*-GFP was used for labelling plant vacuoles [8,12]. Together with the broader pH application range *Pt-*GFP can also be used to monitor pH changes in vacuoles and other acidic compartments or in the cytoplasm under conditions when the cellular environment is switched towards the acidic.

### Mass properties of recombinant pH indicators

The predicted protein masses of *Pt*-GFP and pHluorins are approximately 27 kDa. We confirmed this by denaturating SDS PAGE (data not shown). However, when native proteins were run on FPLC different masses were detected. *Pt*-GFP exhibited a mass of approx. 105 kDa, whereas pHluorins were detected at around 55 kDa. This indicates the formation of dimers in case of pHluorins and of tetramers in case of *Pt*-GFP.

### Cross-sensitivities of recombinant pH indicators

Cross-sensitivities are often major drawbacks when fluorescent proteins are engineered to indicators for cellular signal transduction studies [19,43]. Therefore fluorescence spectra of all three pH-probes were taken *in vitro *with purified protein to check for possible chloride and redox cross-sensitivities. The chloride sensitivities found (Table 3) in the range of 0 < [Cl^-^] < 1 M (at pH = 7.5 in 50 mM Hepes) are negligible (i.e. S << 0.1) and there are also no noteworthy spectral differences between reduced and oxidize probes (i.e. 20 mM DTT in degassed PBS *vs. *50 mM H_2_O_2 _in PBS).

**Table 3:** Cross-sensitivities of recombinant pH-probes. Index 'x' designates sensitivity calculated from excitation spectra, and 'm' calculated from emission spectra

	**GFP-species**		
**parameter**	**ratiometric pHluorin**	**ecliptic pHluorin**	***Pt*-GFP**
S_x _(Chloride)	0.03	0.04	0.04
S_m _(Chloride)	0.02	0.02	0.02
S_x _(red/ox)	0.04	0.05	0.03
S_m _(red/ox)	0.01	0.01	0.02

### In vivo properties of recombinant pH indicators

**Figure 5:**
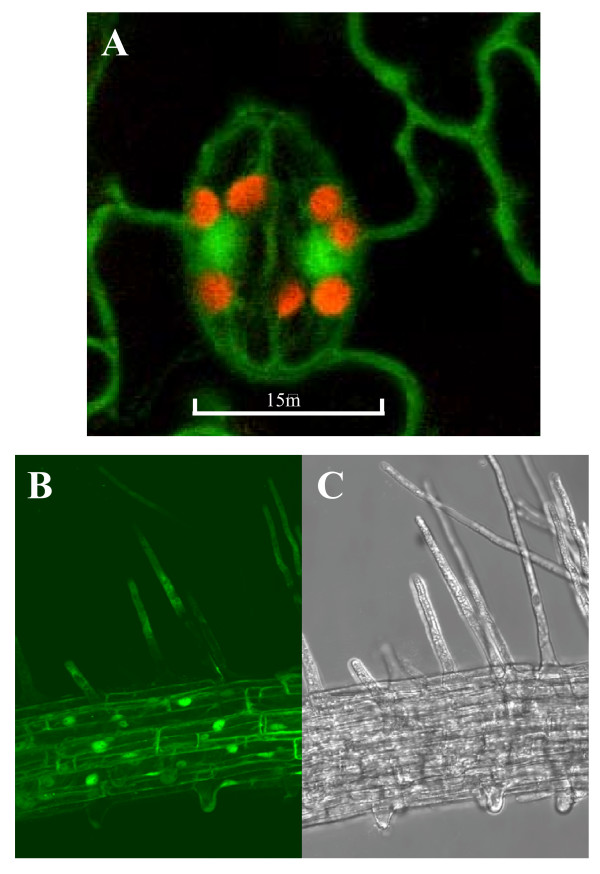
*Arabidopsis *line expressing *Pt*-GFP. **A: **Confocal image of *Arabidopsis *guard cells expressing *Pt*-GFP in the cytoplasm. The red fluorescence is chlorophyll autofluorescence from chloroplasts. **B: **Confocal optical sections and **C: **corresponding bright-field image of a root segment. Excitation with Argon laser line 476 nm; emission at 500–540 nm (green channel) and 600–660 nm (red channel); Leica TCS SP confocal laser scanning system; HC PL APD objective (40× oil).

When cDNA encoding *Pt*-GFP is transferred into the genome of *Arabidopsis *under the control of the CaMV 35S promoter, the fluorescent protein is readily expressed in all cells of the plant and distributes well in the cytoplasm (Figure 5). From *Av-*GFP it has been experienced that this is not a matter of course. First, the *Av-*GFP gene was not entirely accepted by *Arabidopsis *and was expressed only after a cryptic intron was removed from the cDNA [3]. Second, the quantum efficiency was poor in the beginning and mutations were introduced to increase the brightness. Third, the distribution in the cytoplasm was found to be inhomogeneous and further amendments were necessary to increase the cytoplasmic solubility of the protein [4]. Both, the high quantum efficiency of *Pt*-GFP [35] (US Patent No. 6,232,107) and its good expression in plants (Figure 5) do not make any alterations of aminoacid sequence or codon usage necessary.

However, quantum efficiency or brightness of GFPs cannot be directly compared *in vivo *since a lower quantum efficiency could be compensated by a higher expression level. But, the excitation energy or dose is crucial for practical work because it may lead to photodamage or -bleaching when too high. Therefore, we compared exposure times necessary to reach a reasonable signal at the two wavelengths used for *in vivo *ratiometry (Table 4). CCD camera-based *in vivo *ratio imaging systems allow wavelength-independent adjustments of exposure times.

In Figure 2 absorption is normalized by the protein concentration (i.e. by A280). This allows direct comparison of absorption in the visible and demonstrates that *Pt-*GFP has a higher peak absorption here than pHluorins. This promises the need of lower excitation energy. But its very low absorption at 390 nm requires the F390 nm signal having threefold the exposure time of the F475 nm signal to be in the optimal range (Table 4). Fluorescein derivatives like BCECF and FITC have similar asymmetric spectra like *Pt-*GFP and also require appropriate adjustments. For AtpHluorins in contrast such asymmetric adjustment of exposure times is not needed because of the greater symmetry in their spectra. Hence, the higher quantum efficiency of *Pt-*GFP does not necessarily carry forward in a lower excitation energy needed for balanced signals. A way to circumvent this may be to choose instead of 390 nm a wavelength at or closer to the isosbestic point.

### 'In situ'-calibration

**Figure 6:**
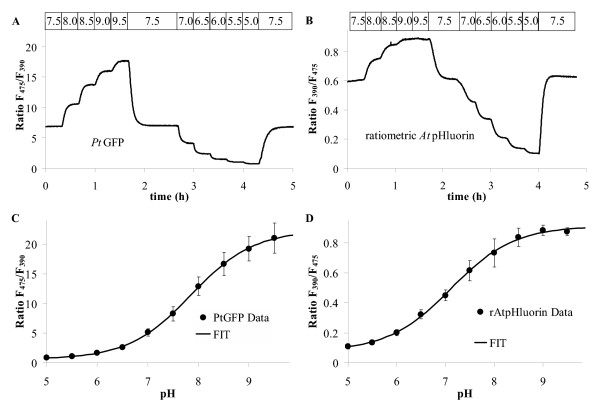
'In situ' calibration procedure. **A, B: **Fluorescence ratio time series of agar beads decorated with *Pt*-GFP (**A**) or ratiometric pHluorin (**B**) during dialysis against buffers of different pH as indicated by the top bar. **C, D: **Resulting calibration curves. Error bars indicate standard deviation. Data in **C **are averages of 9 from 7 individual experiments and data in **D **are averages of 14 from 4 individual experiments.

For converting fluorescence ratio data taken from living cells into pH_cyt _values, an '*in-situ*' calibration procedure was performed using the same optical set-up as for *in vivo *measurements. Therefore, agar beads were decorated with fluorescent protein, sandwiched between sheets of cellophane and dialysed on the microscope against buffers of different adjusted pH (Figures 6A, B). Ratios were plotted over pH and a sigmoidal curve (Boltzmann function) fitted to the data. The low excitation peak of *Pt-*GFP at 390 nm (Figure 1) could be argued to lead to increased variance in the ratio in particular at physiological pH values when F390 is used as denominator. Such spectral imbalance between the two excitation wavelengths used for ratioing is also found with conventional fluorescein derivatives used for pH measurements like BCECF or FITC. However, the errorbars in Figures 6C and 6D show that the scatter of *Pt-*GFP ratios is negligibly increased when compared with ratiometric pHluorin.

Microenvironmental parameters such as viscosity, hydrophobicity, protein mobility, and binding interactions [44] as well as spectral imbalance can be attributed to shifts in the response of an indicator when going from *in vitro *(spectrometer) to *in situ *or to *in vivo *(microscope) recording. Noticeable here is a shift of the apparent pK when comparing Figures 1B and 6C. However, this is not disastrous as long as recordings of calibration data and *in vivo *data are identical.

### pH-clamp

**Figure 7:**
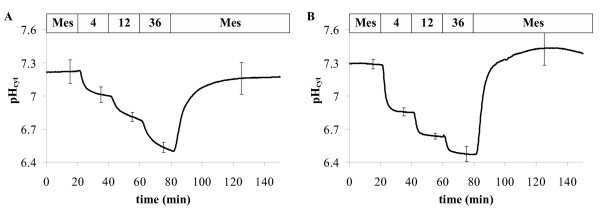
pH-clamp in *Arabidopsis *root cells (hairy zone near the hypocotyl) expressing *Pt*-GFP (**A**) and ratiometric pHluorin (**B**). Perifusionbuffers were MES-buffer (i.e. KCl, CaCl_2_, MgCl_2 _0.1 mM each, 5 mM MES/NaOH pH 5.4) and supplemented with Na-Butyrate of mM-concentrations as indicated by the top bar of the graphs. Curves are averages of 5 from 3 individual experiments. Error bars indicate standard deviation.

The cytoplasmic pH in plants is well buffered [45] and strictly regulated [46]. However, it is possible to adjust cytoplasmic pH values different from the cell's set-point by using weak acids [44,45] or weak bases. Here we performed pH-clamp experiments with *Arabidopsis *expressing *Pt*-GFP and ratiometric pHluorin and recorded the cytoplasmic pH (pH_cyt_) under identical conditions (Figure 7).

### Anoxia – an abiotic stimulus that heavily affects cytoplasmic pH

**Figure 8:**
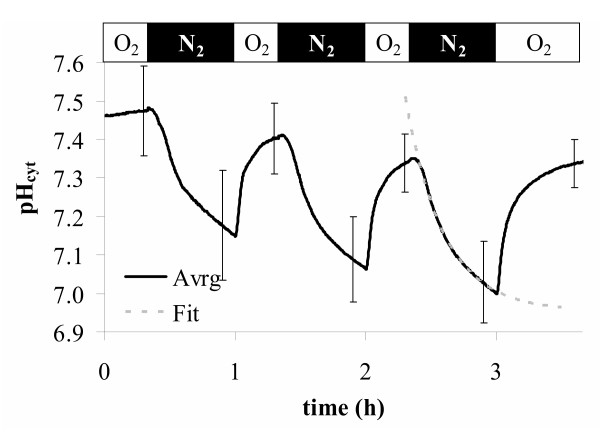
Anoxia-induced pH-deprivation in roots cells (elongation zone near the root tip) of *Arabidopsis *expressing *Pt*-GFP. Given is the average of 7 from 5 individual experiments. Error bars indicate standard deviations. The dotted line gives a first order exponential fit to extrapolate the new predicted steady state pH value (6.95) under anoxia.

Anoxia is a typical abiotic stress factor that appears for instance in clayey or waterlogged soils. Anoxia is known to produce massive acidification of the cytoplasm. Here we demonstrate how *Arabidopsis *expressing *Pt*-GFP report this effect (Figure 8). Cytoplasmic pH shifts in response to low oxygen have been reported many times and quantified by different methods [46,47]. Felle [48] used pH-specific microelectrodes and recorded in *Medicago sativa *under anoxia a fall of pH_cyt _down to 6.8. This is approximately half a pH unit below the normal cytoplasmic level. A shift of similar magnitude was reported from maize roots [49] and from sycamore cells [50] by ^31^P-NMR. The results presented here (Figure 8) match well with the experiments from Felle [48], Ratcliffe [49], and Gout et al. [50].

### Salt stress – an abiotic stimulus that hardly affects cytoplasmic pH

**Figure 9:**
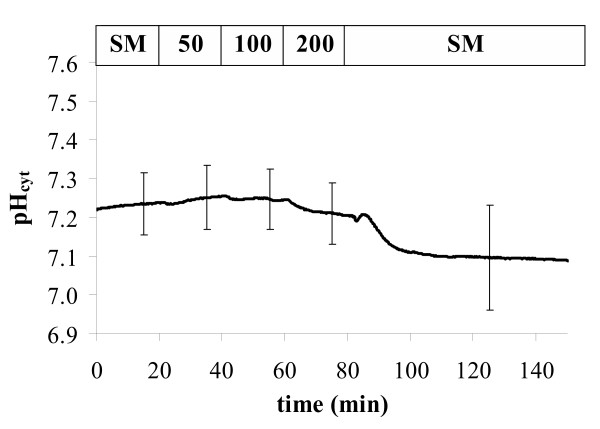
Salt stress-induced pH-changes in roots cells (hairy zone near the hypocotyl) of *Arabidopsis *expressing *Pt*-GFP. Roots were perifused with unbuffered standard medium (SM = KCl, CaCl_2_, MgCl_2 _- 0.1 mM each) and millimolar NaCl concentrations as indicated by the top bar of the graph. Given is the average of 5 from 4 individual experiments. Error bars indicate standard deviations.

Salt stress is a major stress factor leading to massive cuts in crop yield worldwide ([51]; ). One way to combat this problem is to produce crop plants with improved salt tolerance [52-54]. A pre-requisite for this is to understand ion-transport and the mechanisms underlying salt tolerance on cellular and subcellular level. We started to study changes in cellular ion relations under salt stress [21,23]. Here we use *Pt-*GFP expressing *Arabidopsis *to demonstrate how the cytoplasmic pH is affected by increasing concentrations of NaCl (Figure 9). The result coincides well with a similar experiment performed with ratiometric pHluorin [21]. *Arabidopsis *seems to strictly control pH_cyt _when confronted with salt stress. Here (Figure 9) NaCl concentrations above 100 mM leads to slight acidification of approx 0.04 pH units. However, a more pronounced acidification of about 0.2 pH units is observed when saltstress is released. This latter effect can be attributed to H^+ ^coupled Na^+ ^export via Na^+^/H^+ ^antiporter [55]. Other studies on different plant species and conducted with different methods show inconsistent results [55-60]. However, all clearly confirm that salt stress (Figure 9) if at all only slightly (i.e. ΔpH_cyt _< 0.3) affects pH_cyt _when compared to anaerobiosis (Figure 8).

## Summary

Advantages have been found when comparing Pt-GFP with pHluorins:

1 *Pt*-GFP is readily expressed by *Arabidopsis *without any cDNA modifications (Figure 5). Although the cDNA is derived from a distinct and totally unrelated organism, the codon-usage is accepted by *Arabidopsis *and the plant constitutively expresses the protein with high yield under the control of a single 35S-promotor. The use of *Av*-GFP in higher plants, in contrast, was initially limited. Alterations of the codon usage and the removal of a cryptic intron were found necessary to express *Av*-GFP in *Arabidopsis *[3].

2 *Pt*-GFP is readily soluble and distributes well when expressed in the cytoplasm of plants (Figure 5) whereas for *Av-*GFPs modifications where found beneficial to increase protein solubility in the plant cytoplasm [4].

4 The fluorescence excitation ratio of *Pt-*GFP has a maximum dynamic range (R_max _- R_min_) 15 times and a maximum ratio increase (R_max_/R_min_) three times higher than that of ratiometric pHluorin when using excitations at 390 nm and 475 nm (Table 2). It further has a broader area of responsiveness (Figure 3) and thereby also exceeds conventional fluorescein derivatives used for ratiometric *in vivo *pH-measurements (supplemental data in additional file Table S1).

5 *Pt*-GFP is much more robust at low pH (Figure 4). This makes it also suitable for monitoring pH in acidic subcellular compartments or under conditions when the cellular pH is shifted towards the acidic.

Taken together, *Pt*-GFP is an excellent pH indicator for excitation fluorescence ratio imaging and in some respects superior to pHluorins when used in plants.

## Materials and methods

Standard PCR and cloning techniques [61,62] were employed to engineer all constructs described below. cDNAs coding for pHluorins have been cloned and expressed as described [21].

### Bacterial expression, purification, and in vitro-analyses of GFPs

DNA coding for *Pt-*GFP (Acc.No. AY015995) has been subcloned from a pUC18 vector (Nanolight Technologies, Pinetop, AZ, USA) into the bacterial expression vector pRSETb (Invitrogen GmbH; Karlsruhe, FRG). Protein production was induced with 1 mM IPTG when OD600 = 0.6 and expressed at 20°C/300 rpm over night (i.e. 15 h). For protein isolation bacteria were cracked by sonification (HD2200&MS73, Bandelin, Berlin, Germany) in 200 mM phosphate buffer (pH 7.5). Bacterial lysate was pre-cleared at 4,000 × g for 2 h at 4°C. Remaining debris was removed from the supernatant by filtering through a 0.45 μm nylon filter. The 6xHis-tagged fluorescent protein was purified and concentrated through a Ni^2+^/NTA-agarose column (Qiagen, Hilden, Germany). Gel filtration through a NAP-25 column (Pharmacia Biotech, Freiburg, Germany) was performed to remove imidazol from the eluted protein. The purified indicator proteins were assessed spectroscopically. Fluorescence spectra (Figure 1) of the proteins were taken with a fluorescence spectrometer (F-2500, Hitachi) in 150 mM KCl and 50 mM appropriate organic buffers (Mes, Pipes, Hepes, Taps) adjusted to the desired pH with NaOH. Absorption spectra (Figure 2) were taken in phosphate buffer (pH = 7.5) with an absorption spectrometer (2100, Hitachi).

### Size exclusion chromatography (FPLC)

For FPLC protein was bound to a 0.5 ml column of Toyopearl resin (AF-Chelate-650 M; Tosoh Bioscience) washed with Tris/HCl-buffer (50 mM Tris/HCl pH = 8) and treated with Enterokinase (EKMax, Invitrogen) for 24 h at RT. Protein with His-Tag cleaved was washed from the column with 2 ml 1 × PBS and used for fast protein liquid chromatography (FPLC). Briefly, proteins were subjected to FPLC at 4°C with an HiLoad-16/60 Superdex200 column (Amersham Biosciences), equilibrated with 1 × PBS (Medicago AB; Uppsala, Sweden), pre-fitted with a column guard, and driven by a HPLC pump (Äkta-Explorer; Amersham Biosciences) at a flow rate of 1 ml/min. The column was calibrated using a mixture of four proteins of known molecular mass, i.e. catalase (232 kDa), aldolase (158 kDa), chymotrypsinogen A (25 kDa), and ribonuclease A (13.7 kDa).

### Expression in plants

pHluorins for expression in plants were constructed as described in Gao et al. [21]. *Pt*-GFP cDNA (Nanolight Technologies, Pinetop, AZ, USA) was expressed in ecotype Columbia-0 of *Arabidopsis thaliana *under the control of the CaMV 35S promoter using the pART7/pART27 cloning/expression system [63]. Full functionality of *Pt*-GFP in pART7 was assessed by biolistic bombardment and transient expression of young *Arabidopsis *plants (ecotype Col-0) before subcloning the cDNA cassette from pART7 into the binary vector pART27. For agrobacterium-mediated transformation of *Arabidopsis thaliana *(Col-0) the floral dip method [64] was applied.

### Confocal laser scanning microscopy (CLSM)

Transient and stable expressions of GFPs (Figures 6) were assessed by CLSM as described [21] using a Leica TCS SP confocal laser scanning system. For *Pt-*GFP excitation the 476 nm beamline of the Argon laser was chosen; emission at 500–540 nm (green channel) for GFP fluorescence and 600–660 nm (red channel) for chlorophyll autofluorescence; HC PL APD objective (40× oil).

### In situ-calibration of pH probes

For assessment of acid stability (Figure 4) and for in situ-calibration of the pH-indicators (Figure 6) protein was bound to Ni^2+^-agarose beads (Qiagen, Hilden, FRG). Fluorescent beads were sandwiched between two layers of cellulose (Cellophane) and dialysed on the microscope against the buffer solutions indicated in figures.

### In vivo pH-recording

For *in vivo *recording of fluorescence ratios (Figures 7, 8, 9) transgenic *Arabidopsis *were grown in 9 cm Petri dishes on vertical agar as described [65] and used when 6 to 14 days old. Cytoplasmic pH was measured in the hairy root segments near the hypocotyl. Experimental conditions, perifusion technique, and fixation of plant material were described previously [44]. Roots were placed in a volume of 1.6 ml and perifusion flow was adjusted to 2.4 ml/min. The perifused buffer contained KCl, MgCl_2_, and CaCl_2_, 0.1 mM each and 5 mM MES/NaOH adjusted to pH = 5.4. For pH-clamp this buffer was supplemented with different concentrations of sodium butyrate as indicated by the top bar of Figure 7.

### Fluorescence ratio imaging

Fluorescence imaging was performed essentially as described [21,23]. Briefly, fluorescence images at excitation wavelengths of 475 nm and 390 nm were taken every 12 s with a ratio imaging system from TILL-Photonics  fitted to an inverted microscope (Diaphot, Nikon) using light from a monochromator (Polychrome IV, TILL). For the emission path a filter block with beamsplitter 500 dcxr and emission filter HQ535/50 (AHF-Analysentechnik, Tübingen, Germany) was used. TILL software (TILLVision 3.3) was used for processing raw data. The fluorescence ratio F475/F390 was taken with *Pt*-GFP and the ratio (F390/F475) was taken with ratiometric pHluorin as a measure for pH.

### Data analysis

Each spectrum is normalized by its integral (i.e. the sum of fluorescence values over all wavelengths λ). The minimax spectrum F_minimax_(λ) of a set of spectra is determined by substracting the minimal fluorescence from the maximal fluorescence at each wavelength within the obtained set of spectra (i.e. within the scanned analyte concentration range). The isosbestic point (λ_iso_) is determined by looking for the minimum in the minimax spectrum. Ideally, the minimax spectrum is zero at the isosbestic point (i.e. F_minimax_(λ_iso_) = 0). The sensitivity S is defined here by the integral of the minimax spectrum. The sensitivity S of each reporter protein is calculated for the set of its excitation spectra (S_x_) as well as for its corresponding set of emission spectra (S_m_). The Boltzmann fit has been chosen here for fitting sigmoidal curves to calibration data since the Boltzmann equation can directly be derived from the Grynkiewicz equation [66] describing the relation of analyte concentration on fluorescence and fluorescence ratios. The fit parameter of the Boltzmann include R_min_, R_max_, and the apparent pK of the calibrated indicator. Fitting has been performed using Origin 7.0 (OriginLab Corp., Northhampton, MA, USA).

### Availability of materials

Seeds from *Pt-*GFP expressing *Arabidopsis *are freely available from the European Arabidopsis Stock Centre (Nottingham, UK; ). All other novel material described in this studies can be obtained for non-commercial purposes from the corresponding author on request.

## Abbreviations

*Av *= *Aequorea victoria*, *At *= *Arabidopsis thaliana*, CLSM = confocal laser scanning microscopy, DTT = di-Thiotreitol; FPLC = fast protein liquid chromatography, GFP = green fluorescent protein, Hepes = N-2-hydroxy-ethyl-piperazine-N'-2-ethane-sulfonic acid, Mes = 2-[N-morpholino]-ethane-sulfonic acid, PBS = phosphate buffered saline; *Pt *= *Ptilosarcus gurneyi*

## Competing interests

The author(s) declare that they have no competing interests.

## Supplementary Material

Additional file 1 Click here for file
