# Supplementary material for: A novel fluorescent pH probe for expression in plants
Source: Plant Methods. 2006 Apr 6;2:7. doi: 10.1186/1746-4811-2-7 (PMC1475855; doi:10.1186/1746-4811-2-7)
Supplement: Additional file 1 [file 1746-4811-2-7-s1.pdf]

Supplemental material:

Table S1 Spectral characteristics of conventional fluorescent dyes used as pH in vivo indicators. The real isosbestic point is derived from normalized spectra whereas the apparent isosbestic point is from the raw data. The Stokes-shifts are defined here by the difference between the wavelength of the major (and the minor) absorption peak and the wavelength of the emission peak. Index 'x' of S designates sensitivity calculated from excitation spectra, and index 'm' values are calculated from emission spectra. 'em', 'ex', and 'abs' designate wavelengths in the emission spectrum, the excitation spectrum, and in the absorption spectrum, respectively. All data are derived from 10 kDa dextran derivatives:

dextran-BCECF = 2´,7´-Bis(2-carboxyethyl)-5,6-carboxyfluorescein (#D1878, Invitrogen-Molecular Probes); dextran-FITC = Fluorescein-Isothiocyanate (#FD10S; Sigma); dextran-SNARF® = (#D3303, Invitrogen); dextran-NERF® = (#D3319, Invitrogen)

**dye-species** / **parameter**	**BCECF**	**SNARF®**	**FITC**	**NERF®**
real isosbestic point λ_{iso}	489 nm(ex)	610 nm(em)	476 nm (ex)	502 nm (ex)
apparent isosbestic point	439 nm(ex)	626 nm(em)	427 nm (ex)	451 nm (ex)
left peak WL ($\lambda_1 < \lambda_{iso}$)	460nm(ex)	587 nm(em)	454 nm (ex)	472 nm (ex)
right peak WL($\lambda_2 > \lambda_{iso}$)	506nm(ex, abs)	632 nm(em)	495 nm (ex)	514 nm (ex)
Stokes-shift (λ_{em} - λ_{abs})	18 nm	2 nm	21 nm	23 nm
maximum WL (λ_{max})	524 nm(em)	585 nm(ex)	516nm(em)	537 nm (em)
R_{max} ($F(\lambda_1)/F(\lambda_2)$)	4.6	4.9	3.6	3.5
R_{min} ($F(\lambda_1)/F(\lambda_2)$)	0.5	0.25	0.4	0.8
R_{max} - R_{min}	4.1	4.6	3.1	2.7
R_{max} / R_{min}	9.6	19	8.1	4.3
apparent pK (λ_1; λ_2)	6.9	8.7	6.3	5.2
Responsiveness ($\Delta\log(R)\cdot\Delta pH$)	2.2	2.9	1.6	1.2
pH sensitivity (S_x)	**0.9**	0.94	**0.84**	**0.65**
pH sensitivity (S_m)	0.3	**1.0**	0.36	0.36

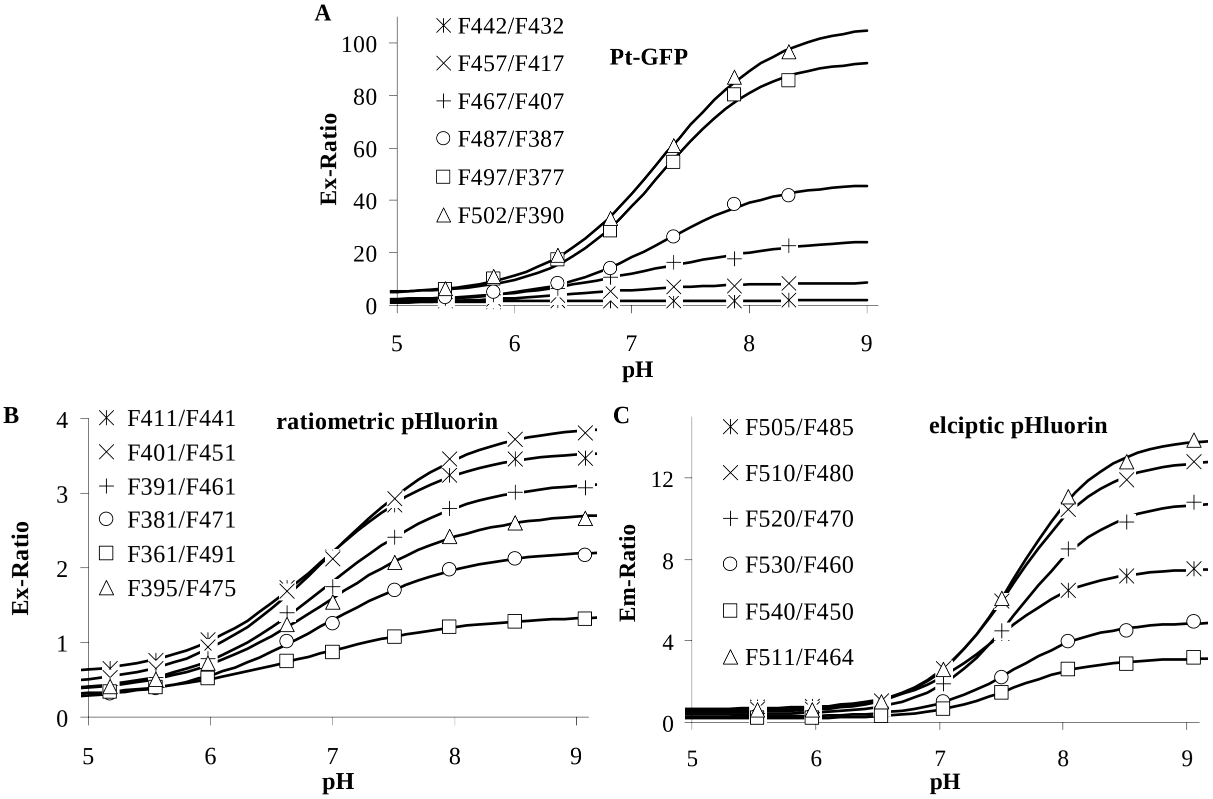

Figure S1 The dependency of ratio calibration curves on the used pair of wavelengths.

Table S2 The dependency of the apparent pK o on the used pair of excitation wavelengths.

GFP	Symbol:	⋇	×	+	○	□	△
***Pt*-GFP**	λ_{ex}-pair	F_{442}/F_{432}	F_{457}/F_{417}	F_{467}/F_{407}	F_{487}/F_{387}	F_{497}/F_{377}	F_{502}/F_{390}
	apparent pK	5.0	6.6	7.1	7.3	7.2	7.3
**ratiometric pHluorin**	λ_{ex}-pair	F_{411}/F_{441}	F_{401}/F_{451}	F_{391}/F_{461}	F_{381}/F_{471}	F_{361}/F_{491}	F_{395}/F_{475}
	apparent pK	6.9	7.0	6.9	6.9	6.8	6.9
**ecliptic pHluorin**	λ_{em}-pair	F_{505}/F_{485}	F_{510}/F_{480}	F_{520}/F_{470}	F_{530}/F_{460}	F_{540}/F_{450}	F_{511}/F_{464}
	apparent pK	7.5	7.6	7.6	7.6	7.6	7.6
